# Clock Genes and Behavioral Responses to Light Are Altered in a Mouse Model of Diabetic Retinopathy

**DOI:** 10.1371/journal.pone.0101584

**Published:** 2014-07-09

**Authors:** Hasna Lahouaoui, Christine Coutanson, Howard M. Cooper, Mohamed Bennis, Ouria Dkhissi-Benyahya

**Affiliations:** 1 INSERM U846, Stem Cell and Brain Research Institute, Department of Chronobiology, Bron, France; 2 University of Lyon, Lyon 1, UMR-S 846, Lyon, France; 3 Laboratory of Pharmacology, Neurobiology and Behavior, URAC-37, University Cadi Ayyad, Marrakech, Morocco; McGill University, Canada

## Abstract

There is increasing evidence that melanopsin-expressing ganglion cells (ipRGCs) are altered in retinal pathologies. Using a streptozotocin-induced (STZ) model of diabetes, we investigated the impact of diabetic retinopathy on non-visual functions by analyzing ipRGCs morphology and light-induced *c-Fos* and *Period 1–2* clock genes in the central clock (SCN). The ability of STZ-diabetic mice to entrain to light was challenged by exposure animals to 1) successive light/dark (LD) cycle of decreasing or increasing light intensities during the light phase and 2) 6-h advance of the LD cycle. Our results show that diabetes induces morphological changes of ipRGCs, including soma swelling and dendritic varicosities, with no reduction in their total number, associated with decreased *c-Fos* and clock genes induction by light in the SCN at 12 weeks post-onset of diabetes. In addition, STZ-diabetic mice exhibited a reduction of overall locomotor activity, a decrease of circadian sensitivity to light at low intensities, and a delay in the time to re-entrain after a phase advance of the LD cycle. These novel findings demonstrate that diabetes alters clock genes and behavioral responses of the circadian timing system to light and suggest that diabetic patients may show an increased propensity for circadian disturbances, in particular when they are exposed to chronobiological challenges.

## Introduction

Diabetic retinopathy is a leading cause of visual impairment and blindness in working-age adults and is expected to continue growing with the increased prevalence of diabetes [1]. Diabetic retinopathy is widely regarded as a microvascular pathology [2] characterized by clinical signs such as neovascularization and macular edema [3]. However, it is currently recognized to be a neurovascular disease with neuronal impairment that precedes vascular damage. This has been documented as electroretinographic abnormalities such as a reduction in a and b-wave amplitude and prolonged latency in the oscillatory potentials in patients with diabetes [4] and in diabetic rodent models [5]. In addition, visual dysfunctions have been reported in rodent models of diabetes [6]–[8] and in diabetic patients that exhibit defects in color perception and impaired contrast sensitivity [9].

These visual abnormalities observed even in the absence of clinical vascular lesions, suggest that diabetes may directly insult the neural retina. Several histopathological studies in diabetic patients [10], [11] and in chemically-induced or genetic diabetic rodent models [12], [13] indicate that retinal ganglion cells (RGCs) are affected by diabetes with apoptotic degeneration. This RGCs degeneration is associated with reduction of the retinal nerve fiber layers [14] and the development of axonal dystrophy [15]–[17].

In the mammalian retina, the melanopsin light sensitive RGCs (ipRGCs) are crucial for the regulation of a range of non-visual functions including the photic synchronization of circadian rhythms [18]–[20]. These cells comprise 1–3% of the total RGCs population and provide photic input to non-visual brain structures, such as the suprachiasmatic nucleus (SCN) that contains the endogenous circadian clock, and the olivary-pretectal nucleus that controls pupillary light reflex.

Loss of ipRGCs leads to circadian, sleep, cognitive and mood disorders [21]. However, the impact of degenerative retinal pathologies on ipRGCs is not well known. In a rat model of glaucoma, we have previously shown a 50–70% reduction of RGCs axon terminals in both visual and non-visual structures including the SCN, accompanied by alterations in the photic behavioral response to light [22]. In addition, rat models of glaucoma and autosomal dominant retinitis pigmentosa show a decrease in the number of ipRGCs [23], [24], a reduction of the pupillary light reflex and of *c-Fos* induction by light in the SCN [23]. Although recent findings evoke a link between circadian dysfunction and diabetes [25]–[27], the effect of retinal degeneration on non-visual functions is still unclear and vary depending on the model of diabetes and species used. In the Ins2^Akita^ genetic mouse model of diabetes, large soma blebs and swelling of ipRGCs are observed, suggesting necrotic-like cell death [17]. In chemically-induced mouse models of diabetes using strepozotocin (STZ) administration, increased melanopsin mRNA and changes in the pupillary light reflex were described [28], whereas in the rat, ipRGCs and pupil response appear to be conserved after 15 weeks post-injection [29].

In this study, we used a chemically-induced STZ mouse model of diabetic retinopathy to investigate the possible impact of retinal degeneration on the circadian timing system during diabetes with a specific focus on alteration of ipRGCs, light-induced clock genes expression in the SCN and photic entrainment of locomotor activity.

## Materials and Methods

### Animals

Wild type male C57BL/6J mice (Janvier, France) were maintained in a temperature-controlled room (23±1°C) under a 12 h light/12 h dark cycle (12L/12D), with food and water ad libitum. All animal procedures were approved by the European Community Council directives and by the regional ethics committee CELYNE (C2EA42-13-02-0402-005). All efforts were made to minimize suffering.

### Induction of experimental diabetes in mice

Diabetes was chemically induced in 3 week-old fasted male mice. Animals received an intraperitoneal injection of STZ (85 mg/kg, Calbiochem, USA) dissolved in 0.01 M sodium citrate buffer (pH 4.5) during 3 successive days [30]. The diabetic state was confirmed by measuring blood glucose levels. Mice with glucose levels higher than 2 g/l were considered to be diabetic. Weight and blood glucose levels were measured at 2, 6 and 12 weeks after the onset of diabetes. Age-matched non-diabetic control animals were injected with 0.01 M sodium citrate buffer.

### Quantification of retinal opsin mRNAs in the retina and light induction of *Per1*, *Per2* and *c-Fos* mRNAs in the SCN

Twelve weeks after the onset of diabetes, mice were maintained under 12L/12D cycle (n = 7 for both groups) and subsequently kept under constant darkness (DD). To analyze *Per1*, *Per2* and *c-Fos* mRNAs induction by light, a pulse of monochromatic light (480 nm, 1.17×10^14^ photons/cm^2^/s; [31]) was administered at circadian time 16 (CT16). After the light pulse, animals were returned to DD and were sacrificed by cervical dislocation 30 min after the beginning of the light pulse. Dark control animals (n = 4–5 for both groups) were handled identically but were not exposed to light. The brains were rapidly removed and under a dissecting microscope, the SCN was localized based on anatomical landmarks and punches taken using thin tweezer. To quantify the expression of short-wavelength (SW), middle-wavelength (MW) opsins, and rhodopsin (Rod) mRNAs, only retinas from the dark control group were used and two retinas from the same animal were pooled. Handling and transfer of the animals and dissections were carried out under dim red light. The SCN and retinas were collected and stored at −80°C until RNA extraction and quantification. Total RNA was extracted using Trizol reagent (Invitrogen) and reverse transcribed using random primers and MMLV Reverse Transcriptase (Invitrogen). Real time RT-PCR was then performed on a Light Cycler system (Roche Diagnostics) using the light Cycler-DNA Master SYBR Green I mix. Hypoxanthine ribosyl-transferase (*Hprt*) was used for normalization. Initially two housekeeping genes were tested for RT-PCR, the *Hprt* and the *36b4* genes. The *Hprt* gene was finally used for internal standardization of target gene expression since *Hprt* only exhibits constitutively, non-regulated expression in both groups and independently of the physiological state and the experimental conditions. This validation was based on the comparison of the crossing points defined as the numbers of PCR cycles required to reach a defined fluorescence intensity between control and STZ-diabetic groups and under different conditions. The efficiency and the specificity of the amplification were determined with a cDNA dilution curve with values between 1.9 and 2.0 and by generating standard curves and carrying out melting curves and agarose gels of the amplicons respectively. Relative transcript levels of each gene were calculated using the second derivative maximum values from the linear regression of cycle number versus log concentration of the amplified gene. Primer sequences were: *Per1* sens, GCGTTGCAAACGGGATGTGT and reverse, GAACCTGCAGAGGTGCCAG; *Per2* sens, GAGTGTGTGCAGCGGCTTA and reverse, ACCAGGTAGGGTGTCATGC; *c-Fo*s sens, AGCGCC CCATCCTTACGGAC and reverse, TCAGCAGATTGGCAATCTCA; *SW* opsin sens, CAGCCTTCATGGGATTTG and reverse, GTGCATGCTTGGAGTTGA; *MW* opsin sens GCTGCATCTTCCCACTCAG and reverse GACCATCACCACCACCAT; *rhodopsin* sens, GCCACCACTCAGAAGGCAG and reverse GATGGAAGAGCTCTTAGCAAAG; *Hprt* sens, ATCAGTCAACGGGGGACATA and reverse, AGAGGTCCTTTTCACCAGCA.

### Immunohistochemistry

Male mice (n = 6 for the control and n = 5 for the diabetic groups) were deeply anesthetized by ketamine (100 mg/kg) and perfused intracardially with saline followed by 4% paraformaldehyde (PFA) in phosphate buffer (PB) at 4°C (pH 7.4) at 12 weeks post-injection.

Flatmounted retinas were rinsed three times (10 min each) in 0.01 M PBS, endogenous peroxidase activity was suppressed using a solution of 50% ethanol with 0.03% H_2_O_2_ for 1 h. Retinas were pre-incubated in 0.01 M PBS, 0.3% triton, 0.1% sodium azide (PBSTA) and blocked with 1% bovine serum albumin (BSA) for 2 h and then in anti-melanopsin primary antibody (1∶2500, Advanced Targeting Systems; UF006) for 2 days, at 4°C, washed twice in PBST, and reacted with the secondary goat anti-rabbit biotinylated IgG (1/200: Vector Laboratories, Burlingame, USA) for 2 h. Immunoreactivity was visualized using a Vectastain ABC Elite kit (PK-6100, Vector Laboratories), followed by incubation in 0.2% 3,3′-diaminobenzidine, 0.5% ammonium nickel sulphate, 0.003% H_2_O_2_ in 0.05 M TRIS buffer (pH 7.6). Flatmounted retinas were mounted on slides, dehydrated in graded ethanol, cleared in xylene and coverslipped. The number and the distribution of melanopsin-positive ganglion cells were determined using the software package Mercator running on ExploraNova technology.

### Light-entrainment of behavioral rhythms

Locomotor activity recording began 3–4 days before mice were injected by STZ. To study the effect of the development of diabetic retinopathy on circadian photoentrainment, we use two light paradigms. In the first experiment, mice (n = 12 for controls and for diabetic) were exposed to 3 successive 12L/12D cycles with stepwise increases of light intensity (from 100 to 300 lux and from 300 to 1000 lux) up to 9 weeks post-injection. At 10-weeks post-injection, mice subsequently underwent a 6 h phase advance of the 12L/12D cycle. The phase angle and the number of days necessary to entrain to the new cycle were determined for each animal. In the second experiment, a second group of animals (n = 12 for controls and for diabetic) were exposed to 12L/12D cycle with decreases of light intensity (from 1000 to 300 lux and from 300 to 100 lux) up to 12-weeks post-injection. During these two light paradigms, different parameters were analyzed: total locomotor activity on 24 h, ratio of night-day activity, onset variability, period using the Chi-squared periodogram and phase angle of entrainment (defined as the time difference between the onset of the activity rhythm and lights OFF of the LD cycle to which the animal is entrained). When the activity onset is in phase with light OFF, the phase angle is around 0 h and was qualified as a normal phase angle in the manuscript. Animals were considered entrained when the onset of their activity rhythms assumed a stable phase relationship relative to the time of lights-OFF for at least 10 days (±0.2 h). The transient effect on the 5 days immediately after each light intensity change was not included in the analysis. During these two light paradigms, the light level was increased or decreased depending on the behavioral parameter of the rhythm of locomotor activity (entrainment and phase angle). Activity records were analyzed with the Clocklab software package (Actimetrics, Evanston, IL).

### Statistical analyses

Two-way ANOVA followed by *post-hoc* Student Newman-Keuls or Fisher were used to compare body weight, blood glucose level, total locomotor activity, phase angle of activity, onset variability, ratio of night-day activity, *Per1-2*, *c-Fos* and opsin mRNAs between control and STZ -diabetic groups. The Mann-Whitney-U test was used to compare the total number of ipRGCs and the number of days necessary to achieve stable entrainment in both groups. Statistical significance was considered for p≤0.05. Values are shown as mean ± SEM.

## Results

### Body weight and blood glucose levels of age-matched control and STZ-diabetic mice

Measurement of blood glucose indicated a significant increase in the STZ-diabetic mice at an early stage of the pathology (2 weeks post-onset of diabetes) and remained elevated at 6 and 12 weeks post-injection (p≤0.001) compared to the age-matched control mice (table 1). In the control group, blood glucose levels remained constant with a mean level of 1.6 g/l. Diabetes also caused a significant loss of body weight compared to the age-matched control animals starting 2 weeks after the onset of diabetes and persisting 12 weeks post-onset of diabetes (p≤0.001).

**Table 1:** Average weight and blood glucose concentrations of control and STZ-diabetic mice.

Duration of	Blood Glucose (g/l)		Body Weight (g)	
diabetes (weeks)	Control	STZ - diabetic	Control	STZ - diabetic
2	1.7±0.1	4.6±0.2***	19.8±0.5	15.9±0.7***
6	1.5±0	3.9±0.5***	24.7±0.8	14.7±1***
12	1.5±0.1	5.2±0.2***	28.1±0.5	20.7±0.7***

Data are expressed as mean ± SEM (n = 15–25 for each group).

***p≤0.001.

### Effect of the diabetic retinopathy on the total number and morphology of ipRGCs

**Figure 1:**
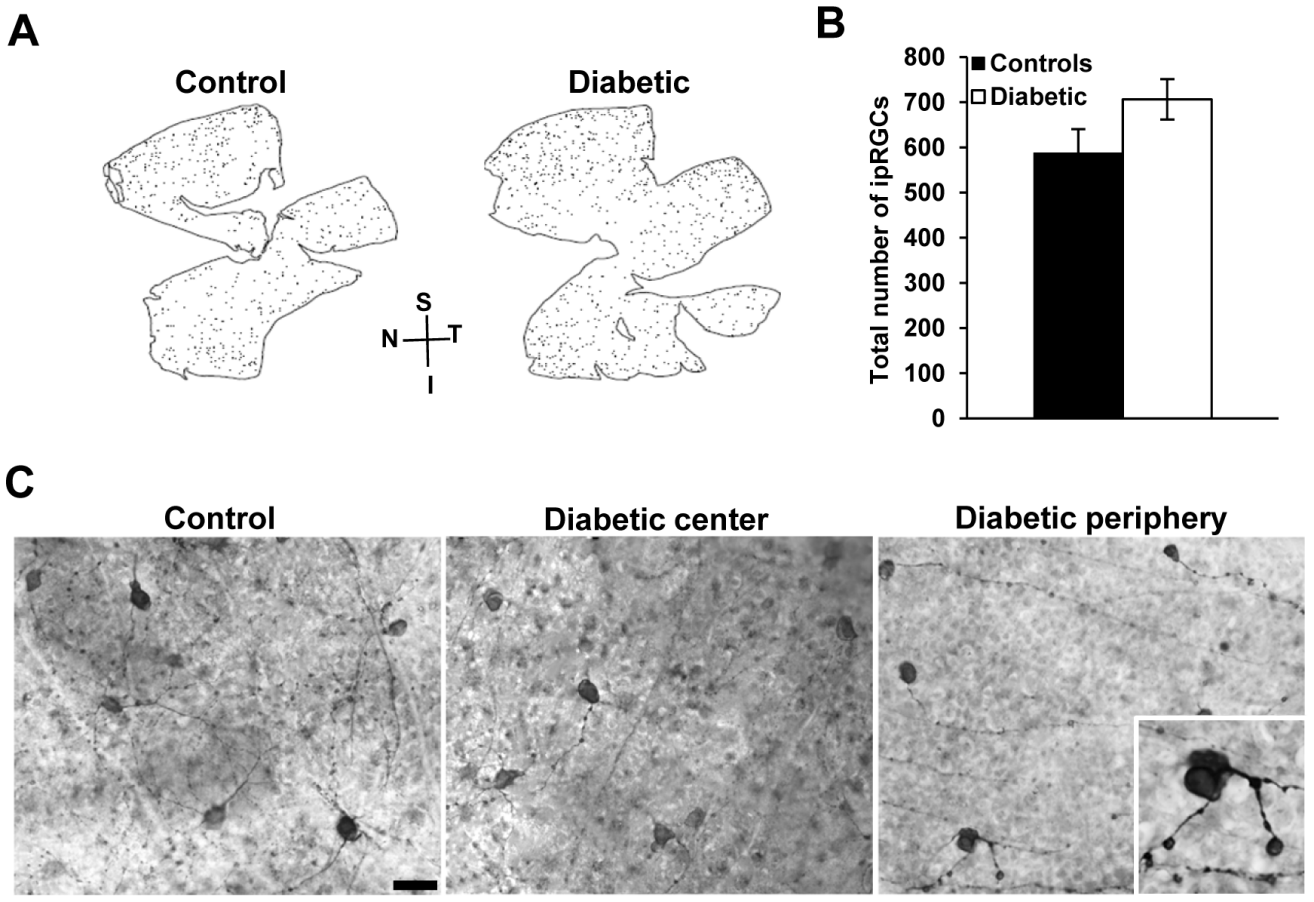
Total number and morphology of ipRGCs in control and STZ-diabetic mice at 12 weeks post-onset of diabetes. (**A**) Representative flat-mounted retinas from a control and a STZ-diabetic animal, immunostained with melanopsin antibody. (**B**) Total number of ipRGCs. No difference was observed between groups. (**C**) Representative photomicrographs of flat-mounted retinas immunostained by melanopsin antibody from control and STZ- diabetic animal at 12 weeks post-diabetes. No morphological abnormalities of ipRGCs were shown in the control retina and in the central retina of STZ-diabetic mouse. Soma swelling and increased varicosities in the dendrites of ipRGCs were noted in the peripheral retina. Enlarged image of a melanopsin-positive cell (Insert: magnification ×100). Data represent means ± SEM (n = 6 for controls and n = 5 for diabetic). Scale bar  = 50 µm. N =  nasal; T =  temporal; S = superior; I = inferior.

The impact of diabetes on ipRGCs was quantified by counting the total number of melanopsin-positive neurons in whole-mounts retinas in STZ-diabetic and age-matched control animals at 12 weeks post-onset of diabetes (Fig. 1A). No significant difference in the total number of melanopsin-positive cells was found between the two groups (controls: 587.6±52.7 cells/retina and STZ-diabetic: 706.4±44.6 cells/retina; p = 0.12; Fig. 1B). The total mean cell densities were also similar (78.04±9 cells/mm^2^ and 76.9±3.5 cells/mm^2^ for control and STZ-diabetic mice respectively). However, in the diabetic mice, ipRGCs showed typical pathological alterations in their morphology. In the control group, ipRGCs somas were round (mean diameter of 10.79±0.35 µm) with a large dendritic field, whereas in the STZ -diabetic group, cells showed large, characteristic blebs, soma swelling (mean diameter of 18.24±0.55 µm) and dendritic varicosities (Fig. 1C and inset). In STZ-diabetic mice, these necrotic-like abnormalities were observed in 6% of the total population of ipRGCs, with a spatial localization essentially in the peripheral retina. None of these abnormalities were detected in controls.

### The functional output of ipRGCs to the SCN is altered in diabetic retinopathy

**Figure 2:**
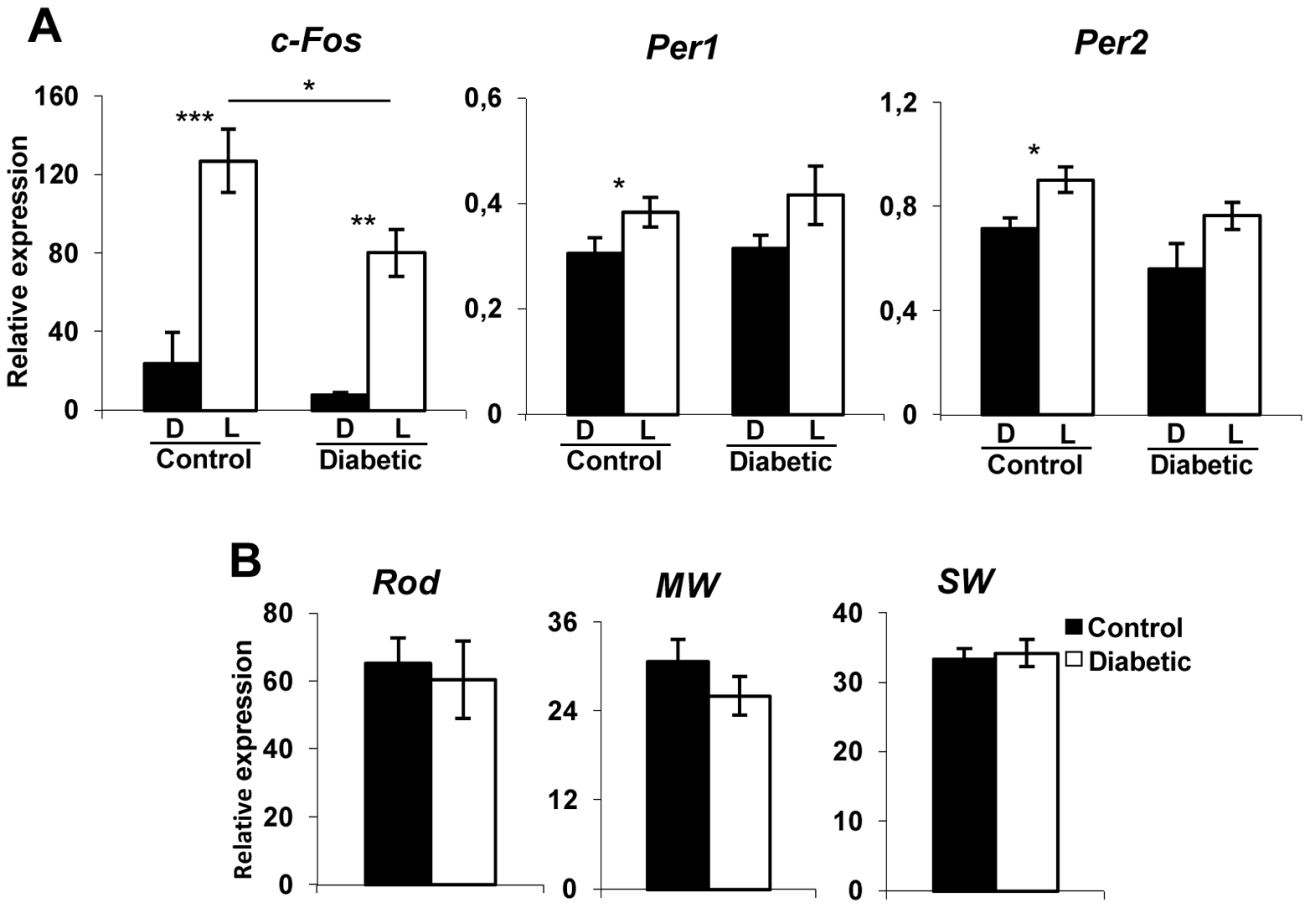
Relative light-induced *c-Fos*, *Per1*, and *Per2* mRNA levels in the SCN and opsin mRNA expression in the retina of control and diabetic mice at 12 weeks post-diabetes. (A) Induction of *c-Fos*, *Per1* and *Per2* in the SCN of control and STZ-diabetic mice by 480 nm monochromatic light (constant irradiance of 1.17×10^14^ photons/cm^2^/s, 15 min) at CT16 using real time RT-PCR. White bars represent animals which received a light pulse (L, n = 7 for each group). Dark controls (D, black bars), were handled in the same way but did not receive a light pulse (n = 4–5 for each group). (B) Relative expression of short wavelength cones (SW), mid-wavelength cones (MW) and rhodopsin mRNAs expression in the retina of the same animals (n = 4–5 for each group). The relative level of opsin mRNAs is equivalent in both groups. Data are presented as means ± SEM. Asterisk indicates a statistically significant difference (*: *p*≤0.05; **: *p*≤0.01 and ***: p≤0.001).

To assess whether these cellular changes may affect the functional output of ipRGCs to the SCN, we analyzed the effect of light on the induction of c-*Fos* and *Period* genes at 12 weeks post-injection. Mice were exposed to a 480 nm monochromatic light pulse of constant irradiance and duration (1.17×10^14^ photons/cm^2^/s; 15 min) at CT16, known to induce significant *Per1-Per2* expression [32], [33]. The proto-oncogene *c-Fos* was significantly induced by light in both control and diabetic mice compared to their respective dark exposed animals (p ≤0.001 for controls and p≤0.01 for STZ-diabetic; Fig. 2A). However, light-induced *c-Fos* was significantly reduced in the STZ-diabetic group compared to the control (p≤0.05). *Per1* and *Per2* mRNAs were both induced in the control group (p≤0.05) whereas no statistically significant induction by light was observed in STZ-diabetic mice (p = 0.22 for *Per1* and p = 0.67 for *Per2*).

Since light transduction is mediated by rods, cones and ipRGCs, we evaluated whether the alteration of responses to light in the SCN of STZ-diabetic mice was related to alteration of rod and cone opsin genes content. Quantification of the expression of SW, MW opsins and rhodopsin mRNAs in the same animals under dark conditions at CT16 revealed no significant difference between the two groups in the relative expression of all retinal opsins tested (Fig. 2B).

### Light entrainment of locomotor activity rhythm is altered in diabetic mice

**Figure 3:**
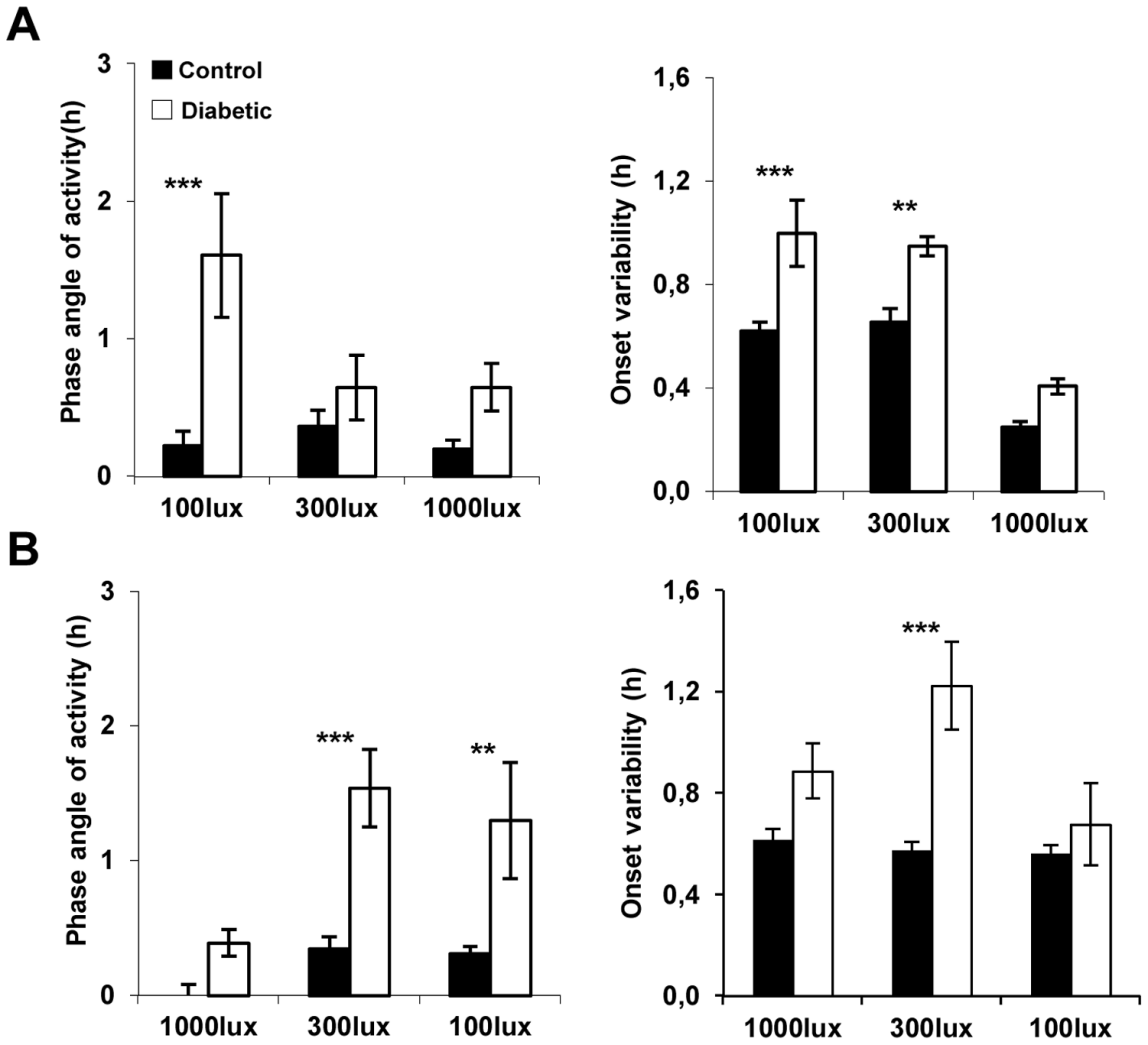
Comparison of the average phase angle of activity and variability onset in control and STZ-diabetic animals submitted to two light schedules. (**A**) Mice were maintained under a 12L/12D cycle and exposed to 3 successive 12L/12D cycle with increases of light intensity (100, 300 lux and 1000 lux; n = 12 for control and for STZ-diabetic mice). Left panel: Phase angle differences between times of lights-off and onset of activity. Positive values indicate an advanced activity onset. At 100 lux, STZ-diabetic mice are entrained with a significant phase advance in relation to the time of lights-OFF compared to the age-matched control animals (p≤0.001). This difference in the phase angle of activity between the two groups is not observed when animals are exposed to 300 lux and at 1000 lux. The activity onsets were also significantly less precise at 100 and 300 lux (right panel). (**B**) In the second light schedule, mice were exposed to 3 successive 12L/12D cycle with decreases of light intensity (1000, 300 lux and 100 lux; n = 12 for control and for STZ-diabetic mice). At 1000 lux, controls and STZ-diabetic animals are entrained with a normal phase angle of activity and with variability around the onset of activity similar to controls (left panel). When the light intensity is decreased (300 and 100 lux), STZ-diabetic animals began their locomotor activity earlier than controls with increased onset variability for STZ-diabetic mice at 300 lux (p≤0.001). Data are presented as means ± SEM, ** p≤0.01 and *** p≤0.001 compared with control.

In order to evaluate the consequences of the development of diabetic retinopathy on the entrainment by light of the circadian system, animals were exposed to two light regimes. In the first paradigm, control and STZ-diabetic animals were subjected to a 12L/12D cycle at three successively increased light intensities during the light phase (100–300–1000 lux), whereas in the second protocol, a decreasing sequence of these light intensities was applied (Fig. 3). These two light paradigms were applied to both groups from the injection of STZ up to 10 weeks post-injection. For both light regimes, the period of locomotor activity rhythms was close to 24 h, with no significant differences between control and STZ-diabetic groups at the three light intensities, indicating the synchronization of the activity rhythm to the 12L/12D cycle (data not shown). When animals were exposed to 12L/12D cycle beginning with low light level (100 lux), STZ-diabetic mice were entrained with a significant phase advance in relation to the time of lights-OFF: 1.6±0.4 h compared to the age-matched control animals 0.2±0.1 h (Fig. 3A; p≤0.001). This difference in the phase angle of activity between the two groups was not observed when animals were exposed to higher light levels of 300 lux (controls: 0.3±0.1 h and STZ-diabetic: 0.6±0.2 h; p = 0.26) and 1000 lux (controls: 0.2 h and STZ-diabetic: 0.6±0.1 h; p = 0.4). The activity onsets were also significantly less precise at 100 and 300 lux, whereas at 1000 lux, no significant difference in the onset variability was observed between the two groups. In both groups, the onset variability decreased from 300 to 1000 lux (p≤0.001; Fig. 3A). When animals were subjected to 1000 lux at the beginning of the experiment, controls and STZ-diabetic animals entrained with a normal phase angle of activity (controls: 0 h; STZ-diabetic: 0.3±0.1 h) and showed similar onset activity variabilities. In contrast, when the light intensity was decreased (300 and 100 lux), STZ-diabetic animals began their locomotor activity earlier than controls (300 lux: controls: 0.3 h; STZ-diabetic: 1.5±0.2 h; p≤0.001; 100 lux: controls: 0.3 h; STZ-diabetic: 1.3±0.4 h; p≤0.01; Fig. 3B) with increased variability onset at 300 lux.

**Figure 4:**
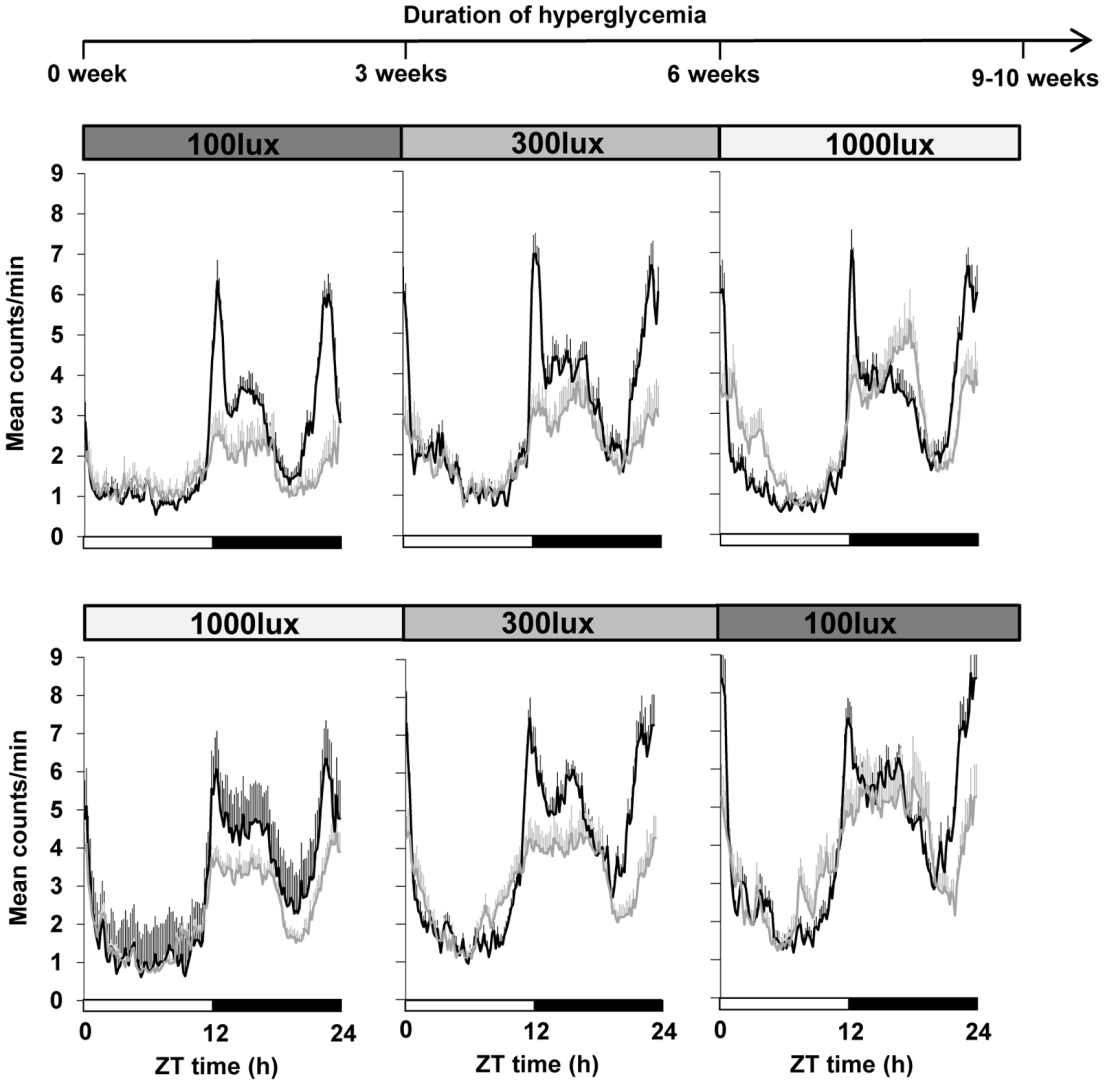
Averaged 24 h locomotor rest-activity profiles of control and STZ-diabetic mice (n = 12 for each group) maintained under 12L/12D cycle. Mice were subjected to 2 different light paradigms. In the first experiment (upper panel), mice were exposed to 3 successive stepwise increases of light intensity (100, 300 lux and 1000 lux) whereas in the second experiment (lower panel), animals were exposed to decreases of light intensity (1000, 300 lux and 100 lux). The first light level (100 or 1000 lux) was applied 3–4 days before the injection of citrate buffer (control group) or STZ (diabetic group) until 3 weeks post-injection. The light levels were then increased (1^rst^ paradigm) or decreased (2^nd^ paradigm) to 300 lux from 3 to 6 weeks post-injection From 6 to 9–10 weeks post-injections, animals were exposed respectively to 1000 or 100 lux (1^rst^ or 2^nd^ light paradigms). Rest-activity profiles of controls (black line) and STZ-diabetic (grey lines) mice were presented from the first day after the 3^rd^ injection of STZ and correspond to 0 week until the end of the protocol (9–10 weeks post-injection). Control animals show a robust daily rhythmicity with a consolidated activity during the dark phase. At all light levels and independently of the light paradigm, STZ-diabetic mice exhibited significantly reduced locomotor activity during the dark phase. Increasing the light levels led to a consolidation of locomotor activity to the dark phase whereas exposing STZ-diabetic mice to decreasing light levels (lower panel) induced a significantly reduced activity during the dark period associated with greater daytime activity. The 12L/12D cycle where represented by the white (light) and the black (dark) bars at the bottom of each panel. Data are expressed as mean count/min averaged across a 10 min bin over around 3 weeks (± SEM).

**Figure 5:**
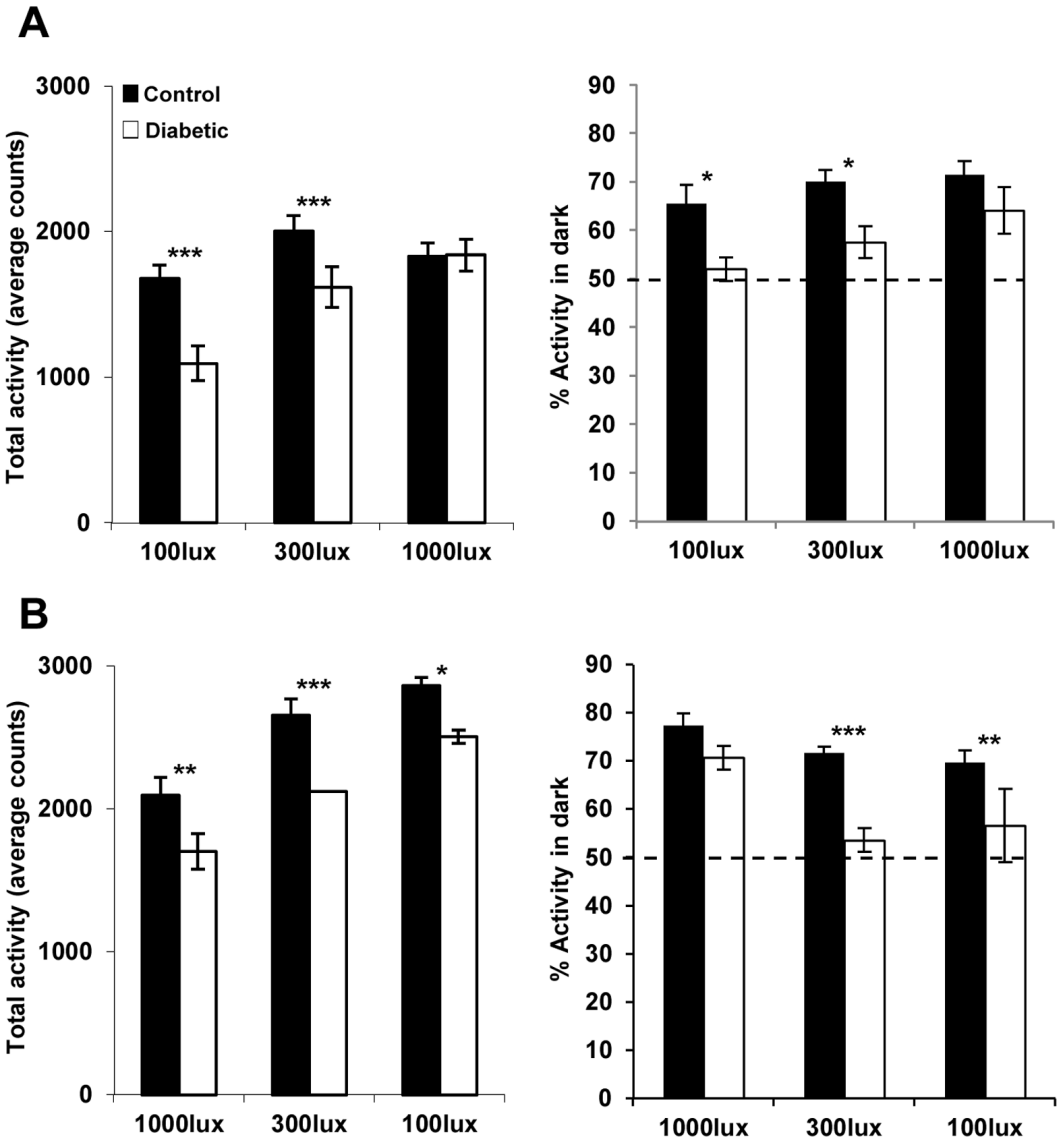
Comparison of the amount of locomotor activity (mean counts per day) and the night-day ratio in control and diabetic animals submitted to two light schedules. (A) Left Panel. Mice were exposed to 3 successive 12L/12D cycle with increases of light intensity (100, 300 lux and 1000 lux). STZ-diabetic mice show a significant reduced locomotor activity at 100 (controls: 1681.9±91.6 counts and STZ-diabetic: 1097.5±119.5 counts; p≤0.001) and 300 lux (controls: 2006±108.2 counts and STZ-diabetic: 1620.6±140.7 counts; p≤0.001), whereas at 1000 lux, no significant difference is shown between the two groups (controls: 1833.2±89.9 counts and STZ-diabetic: 1842±108.5 counts). Right Panel: Percentage of activity during the dark (dotted line  = 50%). STZ-diabetic mice are significantly less nocturnal at 100 and 300 lux. (B) In the second light schedule, animals were submitted to successive decreases in light intensity. These SZT-diabetic mice exhibited a reduction in their total locomotor activity at the three light intensities (at 1000 lux: controls: 2099.5±122.1 counts; STZ-diabetic: 1704.7±125.9 counts; p≤0.01; at 300 lux: controls: 2658.7±112.9 counts; STZ-diabetic: 2124.4±0.1 counts; p≤0.001 and at 100 lux: controls: 2864.7±58.4 counts; STZ-diabetic: 2508.7±47.5 counts; p≤0.05), with a significant decreased ratio of night-day activity at 300 lux and 100 lux. Data are presented as means ± SEM (n = 12 for controls and n = 12 for diabetic for each light schedule), *p≤0.05** p≤0.01 and *** p≤0.001 compared with control.

In the first paradigm (Fig. 4, 5A), the STZ-diabetic group exhibited a significant decrease in the total locomotor activity in comparison to the control group at 100 lux and 300 lux, whereas no significant difference between the two groups was seen at 1000 lux. STZ-diabetic mice were also significantly less nocturnal at 100 lux (ratio of night-day activity of 51.9±2.4% compared to controls 65.5±3.8% p≤0.05) and 300 lux (57.5 ±3.3% compared to controls: 70.1±2.3%, p≤0.05; Fig. 5A). At 1000 lux, STZ-diabetic animals present a significant consolidation of their activity during the dark phase with similar nocturnality (64±4.7%) in comparison to controls (71.4±2.7%). The data shown in Figure 3 suggest a decrease in sensitivity of the circadian system at low light levels. However, it cannot be excluded that the increased age post-injection may also contribute to this effect. To test this hypothesis, another group of control and STZ-diabetic animals was submitted to a second light paradigm, beginning with high light intensity (1000 lux), and then decreased to 300 and 100 lux. The total activity was reduced between control and STZ-diabetic mice at all light levels (Fig. 5B). At 1000 lux, control and STZ-diabetic mice showed similar percentages of nocturnality: 77.4±2.5% for controls and 70.7±2.4% for STZ-diabetic. However, similar to the previous paradigm, when light intensity decreased, the percentage of activity during the dark phase was reduced in the STZ-diabetic mice at 300 lux (controls: 71.7±1.2% and STZ-diabetic: 53.5±2.4%; p≤0.001) and 100 lux (controls: 69.6±2.4% and STZ-diabetic: 56.6±7.6%; p≤0.01) with a slight increase in daytime activity (Fig. 4).

**Figure 6:**
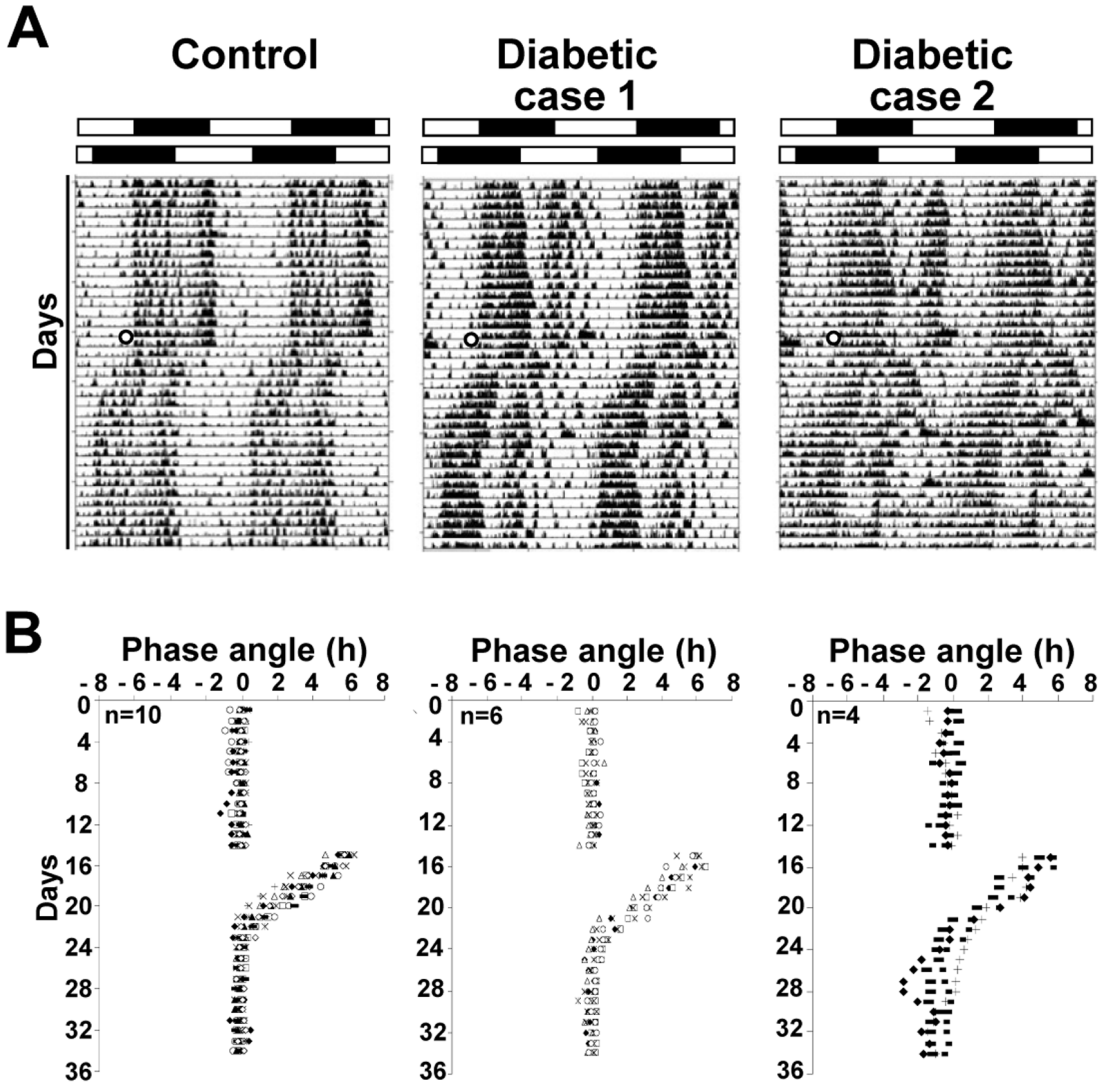
Mean daily activity onsets following a 6/12D cycle for control and STZ-diabetic mice. (**A**) One representative actogram of locomotor activity for control and two for STZ-diabetic mice, double-plotted on a 24 h timescale. The numbered lines represent successive days and the bar above the actograms indicates the original light/dark cycle. After 17 days of entrainment under a 12L/12D cycle at 1000 lux, control and STZ-diabetic mice were exposed to a 6 h advanced light/dark cycle (black dot in the actograms) at 12 weeks post-diabetes. (**B**) Phase angle of photic entrainment. Control mice achieve stable re-entrainment after 8±0.2 days, whereas STZ-diabetic mice need 10.6±0.3 days (Diabetic-case 1; n = 6; p≤0.001). Certain STZ-diabetic mice (n = 4) display a less stable entrainment even after 20 days (Diabetic-case 2). Data are presented as means ± SEM (n = 10 for each group).

At the end of the first light paradigm, when control and STZ-diabetic animals were entrained with a normal phase angle of activity (1000 lux), the capacity of mice to re-entrain to a new 12L/12D cycle was assessed. Fig. 6A shows representative double plotted actograms of control and STZ-diabetic mice before and after the 6 h phase advance of the 12L/12D cycle. Control mice required 8±0.2 days to synchronize to the new light schedule, whereas, 6 STZ-diabetic mice re-entrained, but required an average of 10.6±0.3 days (n = 6; p≤0.001). The 4 other STZ-mice display a less stable entrainment with greater variability during the period of recordings (Fig. 6B).

## Discussion

There is increasing evidence that ipRGCs are subject to alterations in different retinal diseases [22]–[24], [34]–[36]. In diabetic retinopathy, a few studies have reported variable histopathological changes that differ according to model, species, and even within the same species. In the STZ -diabetic mice 12 weeks post-injection, we found that the total number of ipRGCs is not significantly different from age-matched controls. The same result was described in the STZ-rat model after 15 weeks of diabetes onset [29]. However, necrotic-like morphological changes occurred, such as soma blebs and dendritic swellings in 6% of the total ipRGCs population, mainly localized in peripheral retina of the STZ-diabetic mice. This low proportion of atrophic cells may represent one subtype of ipRGCs [37]. The total number of melanopsin-positive cells that we found in the control and the STZ-diabetic mice (around 600–700 cells/mm^2^) is in agreement with prior studies [38], [39] and seems to corresponds to the M1 ipRGCs. In addition, the melanopsin antiserum used in the present study was shown to strongly label the M1 population and weakly the M2 cells [39], [40]. In a genetic model of diabetes, the Ins2^Akita/+^ mouse, melanopsin-immunoreactive cells also show swellings of primary dendrites and increased varicosities on axons after 6 months of diabetes [17]. Taken together, these results indicate that ipRGCs may become progressively impaired as the duration of diabetes increases, as suggested in diabetic patients and mice with altered PLR [28], [34].

Since in previous studies, a surviving population of 10–17% ipRGCs has been shown to be either sufficient or insufficient to maintain non-image forming responses [41]–[43], we evaluated the functional consequences of the atrophic ipRGCs in the STZ-diabetic mouse. Photic activation of ipRGCs leads to induction of the proto-oncogene *c-Fos* and of clock genes *Per1* and *Per2* in the SCN during the night, when light also induces behavioral phase shifts [32]. *c-Fos* is a marker of photosensitivity by comparison to *Per1* and *Per2* genes that are core component of the central pacemaker. In experimental or genetic animal models of diabetic retinopathy, the induction of these genes in response to white or monochromatic light has not previously been examined. Even if the range of induction of *Period* genes in response to the same light intensity is reduced compared to *c-Fos*, we show that *c-Fos* and clock genes induction by a 480 nm light pulse are reduced or abolished at 12 weeks post-onset of diabetes compared to age-matched controls. In controls, the magnitude of *Per1* and *Per2* mRNAs induction by light appears reduced in comparison to previous studies. This discrepancy could be related to the experimental design (30 min stimulation of white light versus 15 min of monochromatic light pulses), the gene assay (*in situ* hybridization versus real time RT-PCR) and/or gene versus protein assay used. Only 3 studies in the STZ and non-insulin-dependent diabetic rat models have evaluated the consequences of diabetic retinopathy on light-induced FOS protein in the SCN [29], [44], [45]. In accordance with our results, they also observed that the number of FOS-immunoreactive cells significantly decreases in the SCN after a white light stimulus (1 h, 100, 300 or 1200 lux) suggesting a deficit in the light induced neuronal activation in the SCN.

The morphological atrophy of ipRGCs may partially account for the defective light input to the central clock. However, since ipRGCs also integrate rod-cone signals and relay this information to non-visual structures [42], it cannot be excluded that the deficits observed are related to alteration of the retinal pathways from outer retinal photoreceptors to the ipRGCs. Previous studies in STZ-induced diabetic rats demonstrate a loss of photoreceptors, the presence of sparse apoptotic cells in the outer nuclear layer and a down-regulation of MW opsin [16], [46], [47]. In the STZ-diabetic rodent model, neuronal degeneration is described to occur predominantly in the inner retina [13], [48]. Indeed, we observed no changes in the expression of mRNA opsins from outer retinal photoreceptors (middle- (MW) and short- (SW) wavelength cones, rods) in the STZ-diabetic mice at 12 weeks post-injection. In addition, we previously showed using the same light protocol (480 nm, 15 min, 10^14^ photons/cm^2^/sec) that the absence of MW-cones does not affect *Per1* and *Per2* mRNAs induction in the SCN [32]. An opacification of the lens has also been evoked to cause a decrease of light sensitivity in the STZ-diabetic rat [29]. Indeed, long-term diabetes mellitus can induce cataract, triggered primarily by the lens aldose reductase activity. However, the mouse lens exhibits a low aldose reductase activity compared to the rat and is extremely resistant to cataract even under sustained hyperglycemia [49], [50] and we did not observe any visible signs of cataract even at 12 weeks post-injection.

The maintenance of an appropriate phase relationship between internal and environmental time has been reported to be dependent on the period of the internal clock as well as the strength of environmental cues. This is the first study that directly assesses entrainment of locomotor activity at different stages during the development of diabetic retinopathy from the injection of STZ up to 10 weeks post-injection. We show that independently of the light level used or the stage of the development of the diabetic retinopathy, STZ-diabetic mice are entrained to the 12L/12D cycle, and behave similarly to control by increasing their total activity with increasing age (from 3 to 6 week-old; p≤0.001). Then, their locomotor activities remain unchanged in both groups until the end of the protocol. However, a number of circadian parameters are altered in the STZ-diabetic group. First, at all light levels and independently of the light paradigm, STZ-diabetic mice exhibited significantly less overall locomotor activity, except at 1000 lux (first paradigm) that can be attributed to a general less healthy condition. Exposure of STZ-diabetic animals to increased light levels led to a consolidation of their locomotor activity to the dark phase without increasing daytime activity (figure 5, right panel). Decreasing the light levels induced a significantly reduced activity during the dark period associated with greater daytime activity as recently described in the STZ rat model [29]. The consolidation of daily rhythms of activity under high light level may be simply explained by the masking inhibitory effect of light on locomotor activity and suggests that STZ-diabetic mice exhibit an unaltered masking response to light [51]. In favor of this, when STZ-diabetic mice were released in DD (data not shown), the onset of their locomotor activity began around the time of lights OFF and was similar to controls.

In addition, STZ-diabetic mice are only capable to entrain to the 12L/12D cycle with a normal phase angle under a high light level that became significantly different when the light intensity decreased, independently of the stage of the pathology. Advanced phase angle with respect to the 12L/12D cycle was previously described for locomotor activity, body temperature and feeding in a genetic model of obesity [52], [53] and in STZ-diabetic rats [54]–[56]. Retinal neurodegeneration, including ipRGCs, is unlikely to occur at an early stage of diabetes [13], when the decreased light sensitivity is already observed in STZ-diabetic mouse. The nature of this disturbance is unknown and can be related to alterations of cell metabolic activity of the retina and/or the SCN itself. Previous studies have shown that changes in brain glucose metabolism attenuate the entraining effects of light on the circadian pacemaker [57] and advance the phase of the rhythmic firing rate of the SCN [58].

In addition to the decrease in circadian sensitivity to light, when STZ-diabetic mice were subjected to a phase advance of the 12L/12D cycle at 12-weeks post-injection, 6 animals re-entrained to the new cycle with a delay, and 4 other individuals exhibited a less stable entrainment in our conditions. Similar deficiencies were described in STZ-diabetic rats [29], [59], but also in other pathological or knockout models such as glaucomatous rats [22] and MW-coneless mice [31].

In conclusion, we show that diabetes alters the light responsiveness of the SCN and the photic entrainment at low light levels and progressively leads to degeneration of ipRGCs. Increasing the light level during the 12L/12D cycle restores normal photoentrainment and consolidates the locomotor activity during the dark phase. These results suggest that patients with diabetes may show an increased propensity for chronobiological disturbances and emphasize the importance of prior light history that have been shown to modulate subsequent light effects on circadian phase shifts, melatonin suppression and ipRGCs sensitivity [60], [61]. Appropriate manipulation of light may be an effective tool to reduce these disturbances in diabetic patients.
